# Associations of concentrations of eight urinary phthalate metabolites with the frequency of use of common adult consumer and personal-care products

**DOI:** 10.1038/s41598-024-55929-2

**Published:** 2024-03-02

**Authors:** Eun A Jang, Kyu Nam Kim, Sang Hyuk Bae

**Affiliations:** 1 Department of Plastic and Reconstructive Surgery, Kangbuk Samsung Hospital, Sungkyunkwan University School of Medicine, Seoul, South Korea; 2 Department of Preventive Medicine, College of Medicine, The Catholic University of Korea, 222, Banpo-Daero, Seocho-Gu, Seoul, 96591 South Korea

**Keywords:** Phthalates, Cosmetics and air fresheners, Urine creatinine, Biological sample, Public health, Quality of life

## Abstract

This study analyzed the relationship between urine concentrations of phthalate metabolites (UCOM) and personal care products (PCPs) used in adults and examined the change in UCOM according to the usage frequency of PCPs based on raw data from the 3rd Korean National Environmental Health Survey conducted between 2015 and 2017. The relationship between PCP use frequency and UCOM was analyzed using multiple regression analysis, adjusting for baseline factors. The regression model consisted of a Crude Model with log-transformed UCOM before and after adjustment for urine creatinine concentrations. Model 1 was additionally adjusted for age, sex, and obesity, while Model 2 was additionally adjusted for smoking, alcohol consumption, pregnancy history, average monthly income of the household, and PCP exposure within the past 2 days. PCP usage frequency was significantly associated with the UCOM without adjustment for urine creatinine and correlated with demographic characteristics, urine creatinine concentration, and PCP exposure within the past 2 days. This study on exposure to urinary phthalates will play a crucial role in Korean public health by aligning with the fundamentals of research priorities and providing representative data on phthalate exposure for conducting population-level studies.

## Introduction

Phthalates such as di-(2-ethylhexyl) phthalate (DEHP), widely known as environmental hormones, constitute a class of endocrine-disrupting compounds^1^. They are color- and odorless liquids added as plasticizers to increase the ductility, durability, and transparency of plastic products widely used in daily life^2^. Particularly, cosmetics, moisturizers, nail polish, adhesives, perfumes, and pesticides frequently used in homes contain a wide variety of phthalates^3,4^. They easily evaporate and are mostly detected in the water or soil after being adsorbed to the water surface owing to wet deposition^5,6^. Typically, the route of exposure is oral, but metabolites in urine following inhalation have also been demonstrated^7^. Phthalates are lipophilic substances that mimic or suppress hormones when absorbed into fat tissues of the body^8,9^. The absorption of phthalates and their metabolites into the body interferes with normal endocrine functions^10^, and European risk assessment data, along with data from the US Environmental Protection Agency (EPA), have indicated that phthalates act as carcinogenic toxins^11^. Typically, the half-lives of phthalates range from 6 to 24 h as they are quickly metabolized in the body and excreted in urine^12,13^. Urine phthalates are biological markers selected as the most common approach to evaluate in vivo phthalates exposure^14^.

Various personal care products (PCPs) are used daily, and their usage frequency varies widely according to age, sex, time of day, and habits. Research on the adverse effects of diethyl phthalate single exposure, intake, and inhalation is almost non-existent. As this substance is widely used in PCPs, exposure via the skin is becoming a significant interest. Despite many overseas studies examining the negative effects of frequent PCP use on health, the harmful effects of PCP ingredients are often underestimated.

Previous overseas studies examining relationships between PCP use and phthalate exposure found that the level of chemical exposure may vary depending on various factors, including country, sex, age, type of cosmetics according to PCP preference, and usage frequency^15^. Recent social trends show that usage of and interest in PCPs is also increasing in men, but prior studies only enrolled women and children while excluding male participants. PCPs have become essential for many people, and their types vary widely. Therefore, different living patterns and socio-environmental factors greatly influence in vivo exposure to phthalates. Phthalates, as chemical compounds used in cosmetics and air fresheners, are subject to investigation by many regulatory agencies worldwide owing to their harmful effects. However, not enough research has been conducted domestically on the relationship between PCP use and phthalate concentrations in the body. Accordingly, this study used raw data from the 3rd Korean National Environmental Health Survey (KoNEHS) to examine the relationship between the use of cosmetics or air fresheners and urine concentrations of phthalate metabolites in Korean adults and to analyze how the frequency of using these products affects the urine concentrations of phthalate metabolites.

## Methods

### Study design

This study used 3rd KoNEHS (2015–2017) data from the National Institute of Environmental Research of the Ministry of Environment. It analyzed the effects of cosmetics and air freshener use on urine concentrations of phthalate metabolites based on data from in vivo measurements of phthalate metabolite concentrations and KoNEHS survey responses regarding demographic and socio-economic factors, residential environment, living habits, eating habits, and environmental exposure of Korean men and women. KoNEHS is a statutory nationwide survey conducted by the National Institute of Environmental Research in accordance with Article 14 of the Environmental Health Act. The survey is designed such that the study participants, selected through sample extraction based on geographic, demographic, and socio-economic distribution, represent the national population.

### Study participants

**Figure 1:**
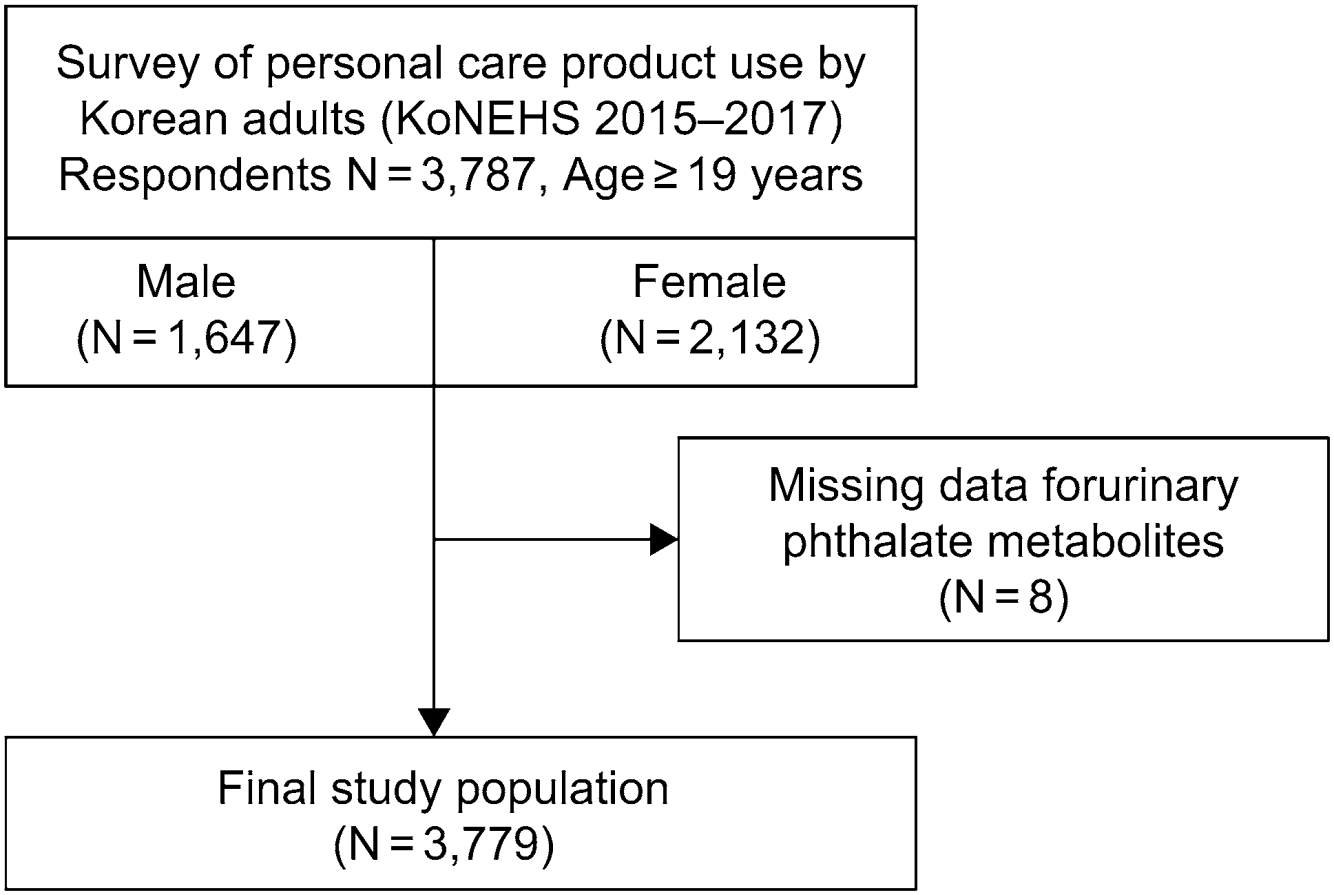
Study flow chart.

The final study population comprised 3779 participants after excluding eight individuals with missing data of urine concentrations (mono-n-butyl phthalate [MnBP], N = 2; monocarboxyoctyl phthalate [MCOP], N = 6) from 3787 adult respondents aged 19 years or older who participated in the 3rd KoNEHS (Fig. 1).

### Study data

The KoNEHS data consisted of responses to a 1:1 interview questionnaire on environmental exposure (163 questions for adults), results of clinical examinations (16 types, including general chemistry), and the assessment of in vivo exposure to environmentally hazardous substances (26 types, including heavy metals). The 3rd KoNEHS was conducted for 3 years to identify the level of exposure to environmentally hazardous substances, such as lead, mercury, and cadmium, in the body (blood, urine) of Korean adults. As the sources of phthalates can vary widely, the results of the questionnaire were grouped by usage frequencies. The survey included a 6-item questionnaire providing information on the use of seven types of PCPs (perfumes, hair products, body cleansers, makeup products, nail polish, antibacterial products, and air fresheners) and the frequency of their use within the past 3 months (once a month, 2–3 times a month, 1–2 times a week, 3–5 times a week, at least 6 times a week, daily); smoking, alcohol consumption, and pregnancy history; average monthly income of the household; and exposure to the products in the past 2 days. Regarding the collection and analysis of biological samples from urine, the data from the biological sample management guidelines and the analysis manual from the 3rd KoNEHS were referenced. The urine concentrations of eight types of phthalate metabolites (mono-(2-ethyl-5-hydroxyhexyl) phthalate [MEHHP], mono-(2-ethyl-5-oxohexyl) phthalate [MEOHP], MnBP, monobenzyl phthalate [MBzP], mono-(2-ethyl-5-carboxypentyl) phthalate [MECPP], MCOP, monocarboxy-isononly phthalate [MCNP], and mono-(3-carboxypropyl) phthalate [MCPP]) were analyzed using liquid chromatography-mass spectrometry. The clinical laboratory test results in the raw data were calculated as the limit of detection (LOD)/√2 (= reporting limit [RL]/√2) or the reporting limit for the substances. The phthalate metabolites present in urine, which are the secondary metabolites of phthalates, are shown in Table 1.

**Table 1:** List of the assessed phthalate metabolites and their parent compounds.

Parent compound	Abbreviation	Urinary metabolite	Abbreviation
Di-(2-ethylhexyl) phthalate	DEHP	Mono-(2-ethyl-5-hydroxyhexyl) phthalate	MEHHP
		Mono-(2-ethyl-5-oxohexyl) phthalate	MEOHP
		Mono-(2-ethyl-5-carboxypentyl) phthalate	MECPP
		Mono-2-ethylhexyl phthalate	MEHP
Di-n-butyl phthalate	DBP	Mono-n-butyl phthalate	MnBP
Benzylbutyl phthalate	BzBP	Monobenzyl phthalate	MBzP
Di-isononyl phthalate	DNP	Monocarboxyoctyl phthalate	MCOP
Di-n-octyl phthalate	DnOP	Mono-(3-carboxypropyl) phthalate	MCPP
Di-isodecyl phthalate	DDP	Monocarboxy-isononly phthalate	MCNP

### Statistical analysis

In this study, weighted 3rd KoNEHS data were analyzed to reflect the complex sample design. Details of the study are as follows.

First, to identify the distribution of the general participant characteristics, the parameters sex, age, obesity, smoking, alcohol consumption, pregnancy history, and average monthly household income were analyzed using descriptive statistics.

Second, the frequencies of cosmetics/air freshener use were scored as follows: Have not used the products in the past 3 months (0); used once a month or less within the past 3 months (1); used 2–3 times a month (2); 1–2 times a week (3); 3–5 times a week (4); used 6 times or more (every day) (5). The relationships of these scores with the urine concentrations of phthalate metabolites were analyzed using multiple regression.

Third, to examine trends in urine concentrations of phthalate metabolites depending on the frequency of cosmetics and air freshener use, relationships of the urine concentrations of phthalate metabolites with the total score (range, 0–25) of frequencies of cosmetics/air freshener use were analyzed using multiple regression.

Among the general characteristics of the study participants presented in the 3rd KoNEHS raw data, the parameters smoking, alcohol consumption, pregnancy history, average monthly household income, and exposure to the products in the past 2 days were calibrated as confounding variables. Since urine concentrations of phthalate metabolites followed a lognormal distribution, these data were log-transformed, whereas the urine concentrations of creatinine are presented as calibrated and uncalibrated values^16^. Statistical significance was defined as p < 0.05, and R Studio (ver 1.4.1106) was used for statistical analysis.

### Ethical approval

All research procedures in this study were performed in accordance with the ethical guidelines of the 1975 Declaration of Helsinki. This study was reviewed by the Institutional Review Board (IRB) of the Catholic University of Korea and obtained exemption approval (study number: MC21ZESE0085) before conducting this analysis. The data used in the study were obtained under the approval of the IRB of the National Institute of Environmental Research. These results are raw data with the participants’ personal information removed, and the anonymity and confidentiality of the participants are guaranteed.

## Results

### General characteristics of the study population

This study analyzed data from 3779 participants (Fig. 1). The numbers of male and female participants were 1647 (43.6%) and 2132 (56.4%), respectively.

The general characteristics of the study participants and their distributions are summarized in Table 2. In the age group 19–29 years, 18.3% were men, and 6.8% were women; between ages 30 and 39 years, 13.6% were men, and 14.9% were women. In the age group 40–49 years, 16.8% were men, and 16.1% were women, showing similar percentages for both sexes. Between ages 50 and 59 years, 21.6% were men, and 24.7% were women; between ages 60 and 69 years, 25.1% were men, and 24.3% were women; and between ages 70 and 86 years, 14.6% were men, and 13.1% were women. Regarding health behavior-related variables, 27.5% of men and 37.7% of women were allocated to the normal BMI group, whereas 72.5% of men and 62.3% of women were classified as obese. Among women, 94.9% had no smoking history, a proportion relatively higher compared to that in men. Former or current smokers accounted for 76.1% of men.

**Table 2:** Demographic characteristics of participants according to sex.

Variables	All participants	Male	Female
Participants, n (%)	3779 (100)	1647 (43.6)	2132 (56.4)
Age (years)			
19–29	281 (7.4)	136 (8.3)	145 (6.8)
30–39	542 (14.3)	224 (13.6)	318 (14.9)
40–49	620 (16.4)	276 (16.8)	344 (16.1)
50–59	882 (23.3)	356 (21.6)	526 (24.7)
60–69	933 (24.7)	414 (25.1)	519 (24.3)
70–86	521 (13.8)	241 (14.6)	280 (13.1)
BMI (kg/m^2^)			
< 18.5	90 (2.4)	29 (1.8)	61 (2.9)
18.5–23	1166 (30.9)	424 (25.7)	742 (34.8)
23–25	962 (25.5)	437 (26.5)	525 (24.6)
≥ 25	1561 (41.3)	757 (46)	804 (37.7)
Smoking			
Never smoker	2417 (64)	394 (23.9)	2023 (94.9)
Former smoker	758 (20.1)	710 (43.1)	48 (2.3)
Current smoker	604 (16)	543 (33)	61 (2.9)
Alcohol consumption			
Never drinker	761 (20.1)	144 (8.7)	617 (28.9)
Drinker	3018 (79.9)	1503 (91.3)	1515 (71.1)
History of pregnancy (present)			
Yes	–	–	2110 (99)
No	–	–	22 (1.0)
Household monthly income (KRW)			
< 1,000,000	711 (18.8)	265 (16.1)	446 (20.9)
1,000,000 to ~ 2,000,000	723 (19.1)	325 (19.7)	398 (18.7)
2,000,000 to ~ 3,000,000	798 (21.1)	364 (22.1)	434 (20.4)
3,000,000 to ~ 5,000,000	935 (24.7)	420 (25.5)	515 (24.2)
5,000,000 to ~ 7,000,000	389 (10.3)	167 (10.1)	222 (10.4)
> 7,000,000	206 (5.5)	99 (6)	107 (5)
Unknown	17 (0.4)	7 (0.4)	10 (0.5)

*Values are presented as numbers (%).

*BMI* body mass index, *KRW* Korean Won.

Men had a higher percentage of drinkers, with 91.3% of men and 71.1% of women being drinkers. Among all female participants, 99% reported a pregnancy history, indicating that a substantially higher percentage of women had a history of pregnancy. The average monthly household income ranged from KRW 3,000,000 to 5,000,000 in 25.5% and 24.2% of men and women, respectively.

### Frequency scores of product use

**Figure 2:**
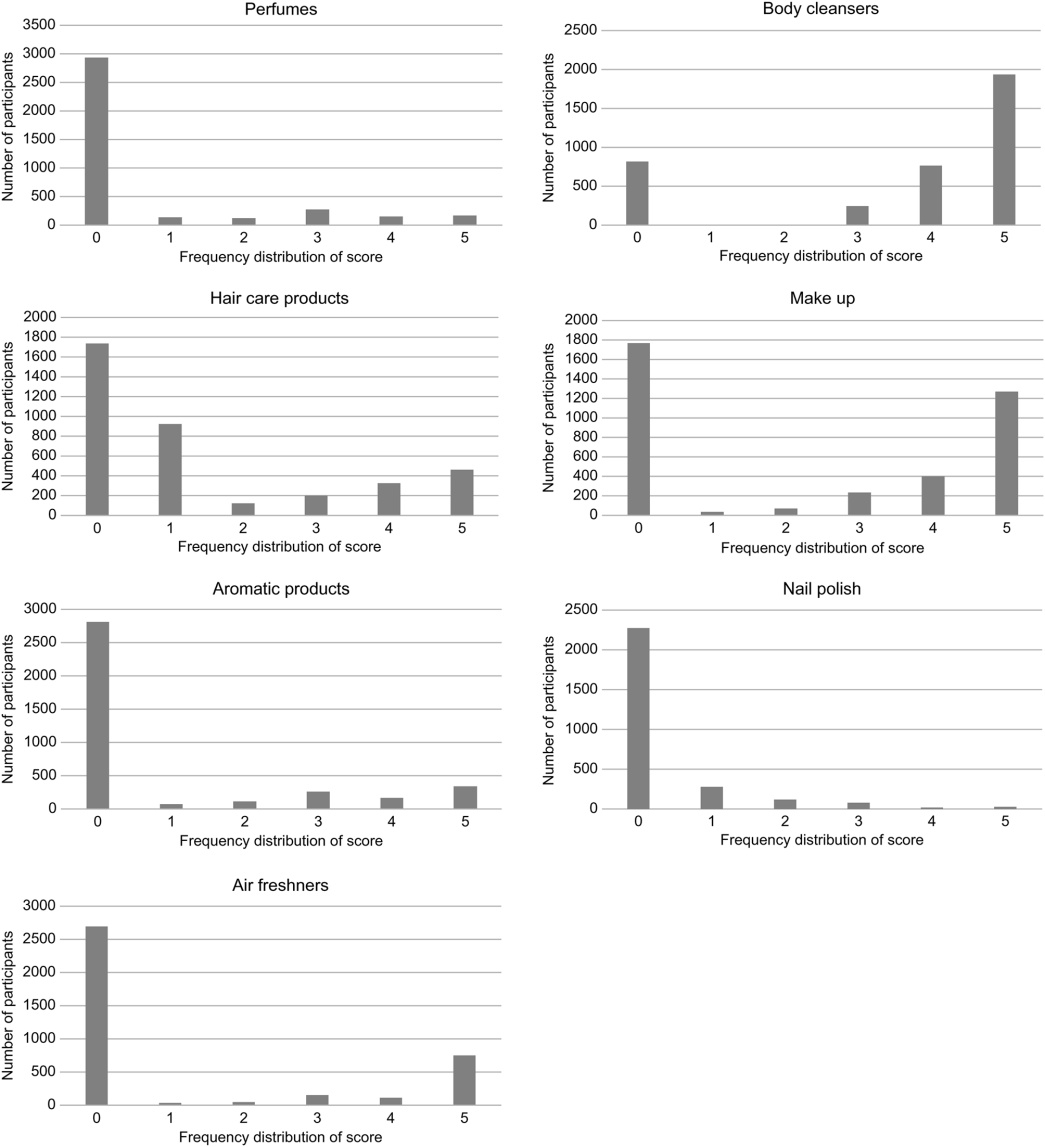
Frequency distributions of the scores.

The usage of each product is presented as a frequency distribution (Fig. 2).

### Multiple regression analysis applying sample weights created through complex sample design

The regression model consisted of a Crude Model with log-transformed urine concentrations of phthalate metabolites before and after adjustment for urine creatinine concentrations. Model 1 additionally adjusted for age, sex, and obesity, while Model 2 additionally adjusted for smoking, alcohol consumption, pregnancy history, average monthly income of the household, and PCP exposure within the past 2 days. The urine concentrations of all DEHP metabolites (∑DEHP) were analyzed using the sum of MEHHP, MEOHP, and MECPP concentrations in urine.

The study findings indicate that the concentrations of DEHP metabolites, i.e., MEHHP, MEOHP, MECPP, and ∑DEHP, were significantly increased in urine in the Crude Model, Model 1, and Model 2 with and without creatinine adjustment according to the frequency of using perfumes, hair products, body cleansers, makeup products, nail polish, antibacterial products, and air fresheners.

Frequent use of perfumes was associated with statistically significant differences (p < 0.001) in MEHHP, MEOHP, MECPP, ∑DEHP, MnBP, and MBzP concentrations in urine after adjustment by creatinine in urine. The increase in the use of perfumes was associated with a statistically significant increase in MECPP concentration (β = 3.581, p < 0.001).

Frequent use of hair products was associated with statistically significant differences (p < 0.001) in MEHHP, MEOHP, MECPP, ∑DEHP, MnBP, and MBzP concentrations in urine after calibration, and the increase in the use of hair products was associated with a statistically significant increase in ∑DEHP concentration (β = 4.121, p < 0.001).

Frequent use of body cleansers was associated with statistically significant differences in MEHHP, MEOHP, MECPP, ∑DEHP, MnBP (all p < 0.001), and MBzP (p = 0.008) concentrations in urine after calibration. The increase in the use of body cleansers was associated with a statistically significant increase in ∑DEHP concentration (β = 4.022, p < 0.001).

Frequent use of makeup products was associated with statistically significant differences (p < 0.001) in MEOHP, MECPP, ∑DEHP, MnBP, and MBzP concentrations in urine after calibration, and the increase in the use of makeup products was associated with a statistically significant increase in ∑DEHP concentration (β = 4.103, p < 0.001).

Frequent use of nail polish was associated with statistically significant differences in MEHHP, MEOHP, MECPP, ∑DEHP, MnBP (all p < 0.001), and MBzP (p = 0.010) concentrations in urine after calibration, and the ∑DEHP concentration (β = 4.207, p < 0.001) showed a statistically significant increase.

Frequent use of antibacterial products was associated with statistically significant differences in MEHHP, MEOHP, MECPP, ∑DEHP, MnBP (all p < 0.001), and MBzP (p = 0.006) concentrations in urine after calibration. The increase in the use of antibacterial products was associated with a statistically significant increase in ∑DEHP concentration (β = 4.098, p < 0.001).

The increase in the use of air fresheners showed statistically significant differences in MEHHP, MEOHP, MECPP, ∑DEHP, MnBP (p < 0.001), MBzP (p = 0.008), and MCOP (p = 0.047) concentrations. The increase in the use of air fresheners was associated with a statistically significant increase in ∑DEHP concentration (β = 4.061, p < 0.001). Only MCOP (β = 0.756, p = 0.047) showed a statistically significant correlation with air freshener use, whereas MCNP and MCPP were not significantly associated with the frequency of any PCP type. The detailed results of the multiple regression analyses are shown in Tables 3 and 4.

**Table 3:** Associations between the frequency of personal care product use and urinary concentrations of phthalate metabolites.

Product	Phthalate metabolite concentration (µg/L)	Crude			Model 1*			Model 2^†^		
		Estimates (β)	SE	p-value	Estimates (β)	SE	p-value	Estimates (β)	SE	p-value
Perfumes	MEHHP	2.655	0.026	< 0.001	2.208	0.223	< 0.001	2.442	0.386	< 0.001
	MEOHP	2.373	0.026	< 0.001	1.987	0.216	< 0.001	1.849	0.425	< 0.001
	MECPP	3.205	0.025	< 0.001	2.854	0.206	< 0.001	3.549	0.389	< 0.001
	∑DEHP	3.959	0.024	< 0.001	3.959	0.024	< 0.001	2.442	0.386	< 0.001
	MnBP	3.167	0.032	< 0.001	2.832	0.288	< 0.001	2.745	0.509	< 0.001
	MBzP	0.785	0.028	< 0.001	0.330	0.202	0.102	0.847	0.472	0.073
	MCOP	0.069	0.022	0.002	0.455	0.188	0.016	0.682	0.430	0.113
	MCNP	− 0.811	0.021	< 0.001	− 0.540	0.158	< 0.001	− 0.621	0.318	0.051
	MCPP	0.145	0.019	< 0.001	0.445	0.445	0.003	0.329	0.311	0.290
Hair care products	MEHHP	2.594	0.032	< 0.001	2.094	0.222	< 0.001	2.680	0.380	< 0.001
	MEOHP	2.298	0.034	< 0.001	1.860	0.217	< 0.001	2.215	0.414	< 0.001
	MECPP	3.180	0.030	< 0.001	2.764	0.203	< 0.001	3.738	0.380	< 0.001
	∑DEHP	3.921	0.029	< 0.001	3.437	0.191	< 0.001	4.162	3.655	< 0.001
	MnBP	3.100	0.040	< 0.001	2.720	0.288	< 0.001	2.943	0.514	< 0.001
	MBzP	0.758	0.036	< 0.001	0.207	0.204	0.310	1.172	0.457	0.010
	MCOP	0.076	0.028	0.006	0.428	0.186	0.022	0.688	0.402	0.087
	MCNP	− 0.829	0.023	< 0.001	− 0.575	0.155	< 0.001	− 0.516	0.311	0.097
	MCPP	0.113	0.023	< 0.001	0.376	0.149	0.012	0.477	0.298	0.109
Body cleansers	MEHHP	2.713	0.049	< 0.001	2.100	0.233	< 0.001	2.600	0.381	< 0.001
	MEOHP	2.435	0.043	< 0.001	1.889	0.221	< 0.001	2.077	0.415	< 0.001
	MECPP	3.203	0.042	< 0.001	2.721	0.207	< 0.001	3.694	0.380	< 0.001
	∑DEHP	3.995	0.040	< 0.001	3.423	0.197	< 0.001	4.113	0.366	< 0.001
	MnBP	3.273	0.056	< 0.001	2.848	0.298	< 0.001	2.921	0.486	< 0.001
	MBzP	0.883	0.048	< 0.001	0.239	0.214	0.266	1.151	0.462	0.013
	MCOP	− 0.052	0.038	0.170	0.343	0.189	0.069	0.711	0.414	0.086
	MCNP	− 0.910	0.038	< 0.001	− 0.660	0.161	< 0.001	− 0.519	0.306	0.090
	MCPP	0.042	0.034	0.222	0.300	0.151	0.046	0.418	0.299	0.162
Make up	MEHHP	2.648	0.034	< 0.001	2.096	0.221	< 0.001	2.563	0.381	< 0.001
	MEOHP	2.343	0.035	< 0.001	1.865	0.217	< 0.001	2.053	0.417	< 0.001
	MECPP	3.222	0.031	< 0.001	2.751	0.202	< 0.001	3.657	0.379	< 0.001
	∑DEHP	3.962	0.030	< 0.001	3.428	0.191	< 0.001	4.078	0.366	< 0.001
	MnBP	3.136	0.044	< 0.001	2.733	0.287	< 0.001	2.940	0.485	< 0.001
	MBzP	0.836	0.036	< 0.001	0.187	0.204	0.359	1.123	0.463	0.015
	MCOP	0.092	0.029	0.001	0.424	0.185	0.022	0.708	0.422	0.093
	MCNP	− 0.802	0.026	< 0.001	− 0.572	0.156	< 0.001	− 0.531	0.308	0.085
	MCPP	0.116	0.026	< 0.001	0.379	0.149	0.011	0.399	0.300	0.183
Nail polish	MEHHP	2.603	0.027	< 0.001	2.225	0.223	< 0.001	2.644	0.383	< 0.001
	MEOHP	2.352	0.028	< 0.001	1.967	0.222	< 0.001	2.123	0.423	< 0.001
	MECPP	3.100	0.029	< 0.001	2.836	0.243	< 0.001	3.850	0.386	< 0.001
	∑DEHP	3.883	0.027	< 0.001	3.513	0.223	< 0.001	4.230	0.372	< 0.001
	MnBP	3.057	0.036	< 0.001	2.958	0.314	< 0.001	3.208	0.489	< 0.001
	MBzP	0.713	0.032	< 0.001	0.313	0.224	0.163	1.285	0.475	0.007
	MCOP	0.033	0.025	0.197	0.548	0.218	0.012	0.902	0.428	0.036
	MCNP	− 0.894	0.024	< 0.001	− 0.490	0.188	0.009	− 0.238	0.313	0.448
	MCPP	0.055	0.021	0.010	0.413	0.170	0.015	0.645	0.307	0.036
Aromatic products	MEHHP	2.579	0.027	< 0.001	2.082	0.223	< 0.001	2.685	0.388	< 0.001
	MEOHP	2.295	0.028	< 0.001	1.851	0.219	< 0.001	2.159	0.420	< 0.001
	MECPP	3.124	0.026	< 0.001	2.723	0.205	< 0.001	3.856	0.386	< 0.001
	∑DEHP	3.883	0.024	< 0.001	3.409	0.193	< 0.001	4.228	0.372	< 0.001
	MnBP	3.098	2.717	< 0.001	2.717	0.291	< 0.001	2.975	0.498	< 0.001
	MBzP	0.710	0.031	< 0.001	0.191	0.206	0.355	1.232	0.463	0.008
	MCOP	0.040	0.023	0.084	0.405	0.187	0.030	0.859	0.413	0.038
	MCNP	− 0.846	0.021	< < 0.001	− 0.596	0.157	< 0.001	− 0.423	0.305	0.166
	MCPP	0.097	0.020	< 0.001	0.358	0.151	0.018	0.561	0.297	0.059
Air fresheners	MEHHP	2.648	0.028	< 0.001	2.183	0.221	< 0.001	2.519	0.442	< 0.001
	MEOHP	2.360	0.029	< 0.001	1.947	0.217	< 0.001	1.871	0.467	< 0.001
	MECPP	3.209	0.027	< 0.001	2.840	0.203	< 0.001	3.667	0.427	< 0.001
	∑DEHP	3.958	0.025	< 0.001	3.516	0.191	< 0.001	4.034	0.418	< 0.001
	MnBP	3.162	0.036	< 0.001	2.808	0.288	< 0.001	2.899	0.531	< 0.001
	MBzP	0.779	0.030	< 0.001	0.303	0.202	0.134	1.081	0.488	0.027
	MCOP	0.098	0.025	< 0.001	0.486	0.187	0.010	0.729	0.410	0.075
	MCNP	− 0.598	0.026	< 0.001	− 0.927	0.183	< 0.001	− 0.454	0.330	0.168
	MCPP	0.149	0.021	< 0.001	0.427	0.150	0.004	0.477	0.310	0.124

*Model 1 adjusted for age, sex, and BMI.

^†^Model 2 adjusted for Model 1 + smoking status, alcohol consumption status, history of pregnancy, household monthly income, and PCP use within the last 2 days.

SE: standard error, ∑DEHP: sum of MEHHP, MEOHP, and MECPP concentrations.

*BMI* body mass index, *DEHP* di-(2-ethylhexyl) phthalate, *MBzP* monobenzyl phthalate, *MCNP* monocarboxy-isononly phthalate, *MCOP* monocarboxyoctyl phthalate, *MCPP* mono-(3-carboxypropyl) phthalate, *MECPP* mono-(2-ethyl-5-carboxypentyl) phthalate, *MEHHP* mono-(2-ethyl-5-hydroxyhexyl) phthalate, *MEOHP* mono-(2-ethyl-5-oxohexyl) phthalate, *MnBP* mono-n-butyl phthalate, *PCP* personal care product.

**Table 4:** Associations between the frequency of personal care product use and creatinine-adjusted urinary concentrations of phthalate metabolites.

Product	Phthalate metabolite concentration (µg/g creatinine)	Crude			Model 1*			Model 2^†^		
		Estimates (β)	SE	p-value	Estimates (β)	SE	p-value	Estimates (β)	SE	p-value
Perfumes	MEHHP	2.840	0.022	< 0.001	1.795	0.189	< 0.001	2.474	0.309	< 0.001
	MEOHP	2.558	0.022	< 0.001	1.573	0.182	< 0.001	1.881	0.335	< 0.001
	MECPP	3.391	0.021	< 0.001	2.440	0.163	< 0.001	3.581	0.300	< 0.001
	∑DEHP	4.144	0.019	< 0.001	4.144	0.019	< 0.001	2.442	0.386	< 0.001
	MnBP	3.352	0.029	< 0.001	2.419	0.255	< 0.001	2.777	0.447	< 0.001
	MBzP	0.970	0.025	< 0.001	− 0.083	0.179	0.643	0.880	0.404	0.030
	MCOP	0.254	0.022	< 0.001	0.041	0.171	0.809	0.714	0.393	0.070
	MCNP	− 0.626	0.025	< 0.001	− 0.954	0.185	< 0.001	− 0.589	0.353	0.095
	MCPP	0.330	0.021	< 0.001	0.031	0.162	0.846	0.361	0.331	0.275
Hair care products	MEHHP	2.757	0.027	< 0.001	1.722	0.187	< 0.001	2.639	0.307	< 0.001
	MEOHP	2.461	0.028	< 0.001	1.489	0.181	< 0.001	2.174	0.325	< 0.001
	MECPP	3.343	0.024	< 0.001	2.393	0.159	< 0.001	3.697	0.294	< 0.001
	∑DEHP	4.084	0.022	< 0.001	3.065	0.146	< 0.001	4.121	0.275	< 0.001
	MnBP	3.263	0.036	< 0.001	2.348	0.254	< 0.001	2.902	0.455	< 0.001
	MBzP	0.921	0.031	< 0.001	− 0.164	0.180	0.362	1.131	0.397	0.004
	MCOP	0.239	0.026	< 0.001	0.057	0.169	0.738	0.647	0.382	0.090
	MCNP	− 0.666	0.028	< 0.001	− 0.947	0.182	< 0.001	− 0.557	0.356	0.118
	MCPP	0.275	0.025	< 0.001	0.005	0.160	0.977	0.436	0.326	0.181
Body cleansers	MEHHP	2.891	0.045	< 0.001	1.723	0.202	< 0.001	2.509	0.307	< 0.001
	MEOHP	2.614	0.039	< 0.001	1.513	0.186	< 0.001	1.986	0.327	< 0.001
	MECPP	3.382	0.038	< 0.001	2.345	0.162	< 0.001	3.603	0.294	< 0.001
	∑DEHP	4.173	0.035	< 0.001	3.047	0.151	< 0.001	4.022	0.276	< 0.001
	MnBP	3.452	0.055	< 0.001	2.472	0.268	< 0.001	2.831	0.428	< 0.001
	MBzP	1.061	0.046	< 0.001	− 0.138	0.191	0.471	1.060	0.397	0.008
	MCOP	0.126	0.039	< 0.001	− 0.033	0.171	0.845	0.620	0.384	0.106
	MCNP	− 0.732	0.047	< 0.001	− 1.037	0.188	< 0.001	− 0.609	0.343	0.076
	MCPP	0.220	0.041	< 0.001	− 0.076	0.162	0.639	0.328	0.323	0.310
Make up	MEHHP	2.701	0.029	< 0.001	1.727	0.187	< 0.001	2.488	0.306	< 0.001
	MEOHP	2.396	0.031	< 0.001	1.495	0.181	< 0.001	1.979	0.326	< 0.001
	MECPP	3.275	0.026	< 0.001	2.381	0.160	< 0.001	3.582	0.290	< 0.001
	∑DEHP	4.077	0.138	< 0.001	3.059	0.146	< 0.001	4.103	0.306	< 0.001
	MnBP	3.188	0.039	< 0.001	2.363	0.252	< 0.001	2.865	0.430	< 0.001
	MBzP	0.889	0.033	< 0.001	− 0.183	0.181	0.313	1.048	0.397	0.008
	MCOP	0.145	0.027	< 0.001	0.055	0.168	0.744	0.633	0.388	0.103
	MCNP	− 0.750	0.031	< 0.001	− 0.942	0.183	< 0.001	− 0.605	0.342	0.077
	MCPP	0.169	0.027	< 0.001	0.010	0.160	0.951	0.324	0.321	0.313
Nail polish	MEHHP	2.794	0.021	< 0.001	1.803	0.169	< 0.001	2.417	0.316	< 0.001
	MEOHP	2.543	0.022	< 0.001	1.545	0.167	< 0.001	1.896	0.340	< 0.001
	MECPP	3.290	0.023	< 0.001	2.414	0.187	< 0.001	3.623	0.301	< 0.001
	∑DEHP	4.205	0.107	< 0.001	3.091	0.163	< 0.001	4.207	0.313	< 0.001
	MnBP	3.247	0.032	< 0.001	2.536	0.269	< 0.001	2.981	0.439	< 0.001
	MBzP	0.903	0.028	< 0.001	− 0.109	0.191	0.569	1.058	0.412	0.010
	MCOP	0.223	0.023	< 0.001	0.126	0.195	0.519	0.675	0.409	0.100
	MCNP	− 0.704	0.027	< 0.001	− 0.912	0.207	< 0.001	− 0.465	0.365	0.203
	MCPP	0.245	0.024	< 0.001	− 0.009	0.185	0.960	0.418	0.348	0.229
Aromatic products	MEHHP	2.797	0.023	< 0.001	1.720	0.189	< 0.001	2.554	0.317	< 0.001
	MEOHP	2.513	0.024	< 0.001	1.489	0.183	< 0.001	2.029	0.334	< 0.001
	MECPP	3.342	0.021	< 0.001	2.361	0.161	< 0.001	3.725	0.298	< 0.001
	∑DEHP	4.260	0.119	< < 0.001	3.047	0.147	< 0.001	4.098	0.280	< 0.001
	MnBP	3.316	0.032	< 0.001	2.355	0.257	< 0.001	2.844	0.443	< 0.001
	MBzP	0.928	0.028	< 0.001	− 0.171	0.182	0.346	1.101	0.399	0.006
	MCOP	0.258	0.022	< 0.001	0.043	0.169	0.797	0.728	0.386	0.059
	MCNP	− 0.846	0.021	< 0.001	− 0.596	0.157	< 0.001	− 0.553	0.348	0.112
	MCPP	0.314	0.022	< 0.001	− 0.004	0.161	0.979	0.430	0.327	0.189
Air fresheners	MEHHP	2.837	0.024	< 0.001	1.772	0.189	< 0.001	2.546	0.359	< 0.001
	MEOHP	2.549	0.025	< 0.001	1.536	0.183	< 0.001	1.898	0.375	< 0.001
	MECPP	3.398	0.022	< 0.001	2.429	0.160	< 0.001	3.694	0.334	< 0.001
	∑DEHP	4.279	0.156	< 0.001	3.105	0.146	< 0.001	4.061	0.321	< 0.001
	MnBP	3.350	0.032	< 0.001	2.397	0.255	< 0.001	2.926	0.472	< 0.001
	MBzP	0.968	0.027	< 0.001	− 0.108	0.180	0.549	1.108	0.414	0.008
	MCOP	0.287	0.024	< 0.001	0.076	0.171	0.661	0.756	0.380	0.047
	MCNP	0.338	0.023	< 0.001	0.016	0.161	0.921	− 0.427	0.359	0.234
	MCPP	0.338	0.023	< 0.001	0.016	0.161	0.921	0.504	0.342	0.140

*Model 1 adjusted for age, sex, and BMI.

^†^Model 2 adjusted for Model 1 + smoking status, alcohol consumption status, history of pregnancy, household monthly income, and PCP use within the last 2 days.

SE: standard error, ∑DEHP: sum of MEHHP, MEOHP, and MECPP concentrations.

*BMI* body mass index, *DEHP* di-(2-ethylhexyl) phthalate, *MBzP* monobenzyl phthalate, *MCNP* monocarboxy-isononly phthalate, *MCOP* monocarboxyoctyl phthalate, *MCPP* mono-(3-carboxypropyl) phthalate, *MECPP* mono-(2-ethyl-5-carboxypentyl) phthalate, *MEHHP* mono-(2-ethyl-5-hydroxyhexyl) phthalate, *MEOHP* mono-(2-ethyl-5-oxohexyl) phthalate, *MnBP* mono-n-butyl phthalate, *PCP* personal care product.

In addition, to further analyze trends in urine concentrations of phthalate metabolites according to the frequency of using any type of PCP, the scores were added for a summary score in the range of 0–25.

The urine concentrations of phthalate metabolites according to the total PCP score were analyzed using multiple regression analysis (Tables 5 and 6). After adjusting for urine creatinine, frequent PCP use was significantly associated with MEHHP, MEOHP, MECPP, ∑DEHP, MnBP, and MBzP concentrations in urine (all p < 0.001). Particularly, ∑DEHP concentrations (β = 4.107, p < 0.001) increased significantly. MCOP, MCNP, and MCPP concentrations were not significantly associated with the total PCP usage score. Comprehensive analysis revealed high usage frequencies of PCPs, and using many PCPs in combination led to statistically significant increases in urine concentrations of phthalate metabolites.

**Table 5:** Associations between the sum of frequencies of PCP use and urinary concentrations of phthalate metabolites.

Variable	Phthalate metabolite concentration (µg/L)	Crude			Model 1*			Model 2^†^		
		Estimates (β)	SE	p− value	Estimates (β)	SE	p− value	Estimates (β)	SE	p− value
Sum of frequencies of PCP use	MEHHP	2.794	0.032	< 0.001	1.889	0.152	< 0.001	2.287	0.314	< 0.001
	MEOHP	2.462	0.034	< 0.001	1.577	0.159	< 0.001	1.963	0.333	< 0.001
	MECPP	3.274	0.030	< 0.001	2.567	0.139	< 0.001	3.489	0.295	< 0.001
	∑DEHP	4.073	0.029	< 0.001	3.232	0.134	< 0.001	3.920	0.285	< 0.001
	MnBP	3.230	0.042	< 0.001	2.635	0.195	< 0.001	2.712	0.409	< 0.001
	MBzP	0.994	0.039	< 0.001	0.129	0.183	0.480	1.071	0.388	0.006
	MCOP	− 0.037	0.027	0.162	0.207	0.125	0.099	0.557	0.270	0.039
	MCNP	− 0.892	0.024	< 0.001	− 0.735	0.114	< 0.001	− 0.461	0.242	0.057
	MCPP	0.040	0.023	0.076	0.079	0.107	0.462	0.320	0.224	0.153

*Model 1 adjusted for age, sex, and BMI.

^†^Model 2 adjusted for Model 1 + smoking status, alcohol consumption status, history of pregnancy, household monthly income, and PCP use within the last 2 days.

SE: standard error, ∑DEHP: sum of MEHHP, MEOHP, and MECPP concentrations.

*BMI* body mass index, *DEHP* di-(2-ethylhexyl) phthalate, *MBzP* monobenzyl phthalate, *MCNP* monocarboxy-isononly phthalate, *MCOP* monocarboxyoctyl phthalate, *MCPP* mono-(3-carboxypropyl) phthalate, *MECPP* mono-(2-ethyl-5-carboxypentyl) phthalate, *MEHHP* mono-(2-ethyl-5-hydroxyhexyl) phthalate, *MEOHP* mono-(2-ethyl-5-oxohexyl) phthalate, *MnBP* mono-n-butyl phthalate, *PCP* personal care product.

**Table 6:** Associations between the sum of frequencies of PCP use and creatinine-adjusted urinary concentrations of phthalate metabolites.

Variable	Phthalate metabolite concentration (µg/g creatinine)	Crude			Model 1*			Model 2^†^		
		Estimates (β)	SE	p-value	Estimates (β)	SE	p-value	Estimates (β)	SE	p-value
Sum of frequencies of PCP use	MEHHP	2.962	0.028	< 0.001	1.606	0.124	< 0.001	2.423	0.247	< 0.001
	MEOHP	2.630	0.030	< 0.001	1.293	0.132	< 0.001	2.099	0.272	< 0.001
	MECPP	3.440	0.025	< 0.001	2.283	0.113	< 0.001	3.625	0.237	< 0.001
	∑DEHP	4.210	0.024	< 0.001	3.159	0.108	< 0.001	4.107	0.222	< 0.001
	MnBP	3.399	0.037	< 0.001	2.352	0.171	< 0.001	2.848	0.359	< 0.001
	MBzP	1.161	0.034	< 0.001	− 0.154	0.157	0.327	1.207	0.332	< 0.001
	MCOP	0.129	0.026	< 0.001	− 0.077	0.121	0.528	0.693	0.261	0.008
	MCNP	− 0.726	0.029	< 0.001	− 1.019	0.135	< 0.001	− 0.325	0.288	0.260
	MCPP	0.207	0.025	< 0.001	− 0.205	0.113	0.069	0.456	0.239	0.057

*Model 1 adjusted for age, sex, and BMI.

^†^Model 2 adjusted for Model 1 + smoking status, alcohol consumption status, history of pregnancy, household monthly income, and PCP use within the last 2 days.

SE: standard error, ∑DEHP: sum of MEHHP, MEOHP, and MECPP.

Sum of frequencies of using PCPs: less than once a month within the last 3 months, once a month within the last 3 months, twice or three times a month within the last 3 months, once or twice a week within the last 3 months, three or five times a week within the last 3 months, six times a week and more within the last 3 months.

*BMI* body mass index, *DEHP* di-(2-ethylhexyl) phthalate, *MBzP* monobenzyl phthalate, *MCNP* monocarboxy-isononly phthalate, *MCOP* monocarboxyoctyl phthalate, *MCPP* mono-(3-carboxypropyl) phthalate, *MECPP* mono-(2-ethyl-5-carboxypentyl) phthalate, *MEHHP* mono-(2-ethyl-5-hydroxyhexyl) phthalate, *MEOHP* mono-(2-ethyl-5-oxohexyl) phthalate, *MnBP* mono-n-butyl phthalate, *PCP* personal care product.

## Discussion

This study was conducted using data from the 3rd KoNEHS to examine the effects of cosmetics and air freshener use on urine concentrations of phthalate metabolites. According to the statistical information report from KoNEHS (2020)^17^, in vivo phthalate exposure continuously decreases in adults. However, if domestic and overseas regulations for hazardous substances differ, domestic products may experience reduced competitiveness or be impacted by administrative actions from the exporting country or corrective measures such as a product recall. Furthermore, importing products from other countries containing these hazardous substances may result in domestic environmental pollution and health hazards. Therefore, environmental hazards are continuously discussed by reflecting analyses of overseas samples, the infrastructure for analysis, socio-economic issues, and chemical distribution volumes. Accordingly, it is necessary to analyze the correlation between the use of cosmetics and air fresheners and urine concentrations of phthalate metabolites in the long term^17^.

Previous overseas studies identified correlations between urine concentrations of phthalates and various household goods or PCPs, confirming that the exposure to phthalates increases according to the usage of these products^18,19^. Many previous studies conducted multiple regression analyses by applying standard weights created via complex sample design after logarithmic conversion as the urine concentration of phthalate metabolites followed lognormal distributions^3,4,19–21^. These analysis methods of previous studies were referenced in our study to identify the correlation between PCP usage frequency and phthalate metabolite measurements in biological samples.

In this study, analyzing urine samples and questionnaire responses from Korean adults, increases in PCP use led to statistically significant increases in urine concentrations of phthalate metabolites. To determine their correlation with urine concentrations of phthalate metabolites, PCP usage frequencies were classified into five categories. Although this scoring approach differs from methods of previous studies reporting associations between cumulative PCP use and phthalate urine concentrations^18,22^, our study results confirmed that the summary score reflecting PCP usage was significantly associated with urine concentrations of phthalate metabolites.

In this study, the participants’ sex, age, obesity, smoking, alcohol consumption, pregnancy history, average monthly household income, and exposure to PCPs within the past 2 days were incorporated into the model as confounding variables. Previous studies confirmed a positive correlation between obesity and urine concentrations of phthalate metabolites^23–25^. Smoking history and the daily number of cigarettes smoked affect lung function; a previous study confirmed that smoking history is positively correlated with monobutyl phthalate (MBP) concentrations among phthalates^26^. Prior research confirmed the correlation between PCP use in women of childbearing age or pregnant women and urinary phthalate concentrations^18,27^. According to a study by the National Health and Nutrition Examination Survey (NHANES) (2015–2016), average monthly household income positively correlated with phthalate concentrations.^28,29^

In assessing short-term DEHP exposure, the urine concentration of this phthalate has a particular meaning in our study. The urine concentrations of DEHP metabolites, including MEHHP, MEOHP, MECPP, and ∑DEHP, were significantly associated in all three models before and after adjustment for urine creatinine concentrations with the frequencies of using perfumes, hair products, body cleansers, makeup products, nail polish, antibacterial products, and air fresheners as determined by the questionnaire.

Many previous studies have reported that people who use PCPs are exposed more to phthalates than those who do not use them^3,30–34^. For instance, phthalates were detected in higher amounts in leave-on products such as nail polish, perfumes, and antibacterial products than in rinse-off products^30^. This finding aligns with our study finding that, except for MCOP, MCNP, and MCPP, urine concentrations of phthalate metabolites (β) increased with higher frequencies of nail polish, perfume, and antibacterial product use. In previous studies, the use of perfumes or air fresheners was highly correlated with urine concentrations of mono-isobutyl phthalate (MIBP), DEHP, MEOHP, and MEHHP^19^. Similarly, statistically significant differences were observed in our study for secondary DEHP metabolites, including MEHHP and MEOHP. Women who frequently use makeup products throughout most of the week had high MBzP levels^19^, which is consistent with our finding that the frequency of makeup product use was significantly associated with MBzP concentrations. Previous studies found that the use of eye makeup products is highly correlated with MBP, MBzP, MIBP, and MEOHP concentrations^19^, but this could not be verified in our study as the KoNEHS questionnaire did not subclassify makeup products. The use of nail polish was highly correlated with MnBP concentrations in previous studies^19^ but with ∑DEHP and MECPP concentrations in this study.

In conclusion, although we hypothesized correlations between particular PCP groups and urine concentrations of specific phthalates, we found either no correlation or correlations consistent with previous findings.

The limitations of this study are as follows. First, as the study analyzed KoNEHS raw data, causal relationships cannot be established; however, correlations between the frequency of PCP use and urine concentrations of phthalates were identified.

Second, PCP types included in the KoNEHS questionnaire were fragrance, hair care products, body cleansers, makeup, nail polish, and air fresheners. In contrast, deodorant and spray were not included in the KoNEHS questionnaire used in this study. Furthermore, unknown factors other than PCPs may have influenced the urine concentrations of phthalate metabolites. In addition, products often advertised as “natural,” “odorless,” and “without added chemicals” may still contain phthalates to eliminate odors^35,36^. The routes of exposure to phthalates vary widely, which means they could not be investigated in our study and might be confounding variables.

Third, previous studies conducted overseas have assessed PCPs and urine concentrations of phthalate metabolites in detail; however, the items listed in the questionnaire used in our study were limited in number. Further research with a more detailed categorization of PCPs and phthalate metabolites should be conducted in future studies.

Fourth, phthalates have a short half-life and are metabolized quickly in the body and excreted^12^, which means that the exposure is reflected by urinary concentrations only for a certain time. The half-life of DEHP is approximately 1 day^13^. Taking this into consideration, the exposure to PCPs within the past 2 days in the KoNEHS questionnaire was adjusted for confounders. However, the causal relationship with the use of products at the time of exposure remains unclear, as the exposure was reflected by a transient increase in urine concentrations.

However, KoNEHS is significant because it is a bio-monitoring survey with nationwide samples representative of the entire country, surveying the concentration of environmentally hazardous substances in Korean adults. In this study, accurate information on PCP use and phthalate quantities could not be obtained, but considered together with the results of previous studies, the secondary metabolites of phthalates contained in PCPs could be identified. Furthermore, our study findings verified significant associations between the frequent use of different types of PCPs and urine phthalate metabolites. Based on the results of this study, we intend to explore the specific relationship between certain phthalates and PCPs in future studies. For example, although the independent variables were PCPs and dependent variables were phthalate metabolites in our study, the variables will be reversed in subsequent studies, with independent variables as phthalate metabolites and dependent variables as PCPs. This reversal can help derive more scientifically correct correlations between them.

This study demonstrates that increased use of PCPs and phthalate exposure increases the levels of several urinary phthalate metabolites. After adjusting for age, sex, BMI, smoking status, alcohol consumption status, history of pregnancy, household monthly income, and using PCPs within the last 2 days, they were correlated with urinary phthalate metabolites. The levels of several phthalate metabolites in urine were positively correlated with the numbers using PCPs. A negative association was observed between the level of MCNP, MCPP, and the use of PCPs. Additionally, a wide variety of PCPs were also related to urinary phthalate metabolites, of particular interest, fragrance, nail polish, and aromatic products, posing a threat owing to their residues, which had significantly higher concentrations of MEHHP, MEOHP, MECPP, ∑DEHP, MnBP and MBzP than rinse-off products. This study on exposure to urinary phthalates will serve an important role in Korean public health by aligning with the fundamentals of research priorities and indicating representative data on phthalate exposure with which population levels can be determined.

## Data Availability

The datasets used and analyzed during this study are available from the corresponding author upon reasonable request.
